# Genetic Differentiation of the Mitochondrial Cytochrome Oxidase *c* Subunit I Gene in Genus *Paramecium* (Protista, Ciliophora)

**DOI:** 10.1371/journal.pone.0077044

**Published:** 2013-10-29

**Authors:** Yan Zhao, Eleni Gentekaki, Zhenzhen Yi, Xiaofeng Lin

**Affiliations:** 1 Laboratory of Protozoology, Institute of Evolution & Marine Biodiversity, Ocean University of China, Qingdao, China; 2 Laboratory of Protozoology, College of Life Science, South China Normal University, Guangzhou, China; 3 Department of Biochemistry & Molecular Biology, Dalhousie University, Halifax NS, Canada; University of Texas Medical School at Houston, United States of America

## Abstract

**Background:**

The mitochondrial cytochrome *c* oxidase subunit I (*COI*) gene is being used increasingly for evaluating inter- and intra-specific genetic diversity of ciliated protists. However, very few studies focus on assessing genetic divergence of the *COI* gene within individuals and how its presence might affect species identification and population structure analyses.

**Methodology/Principal findings:**

We evaluated the genetic variation of the *COI* gene in five *Paramecium* species for a total of 147 clones derived from 21 individuals and 7 populations. We identified a total of 90 haplotypes with several individuals carrying more than one haplotype. Parsimony network and phylogenetic tree analyses revealed that intra-individual diversity had no effect in species identification and only a minor effect on population structure.

**Conclusions:**

Our results suggest that the *COI* gene is a suitable marker for resolving inter- and intra-specific relationships of *Paramecium* spp.

## Introduction

The ciliated protozoa comprise one of the most ecologically important microbial eukaryotic groups [1]. Their morphology is extremely diverse encompassing a multitude of shapes and sizes [2]–[4]. Initially, diversity studies of ciliates were focused exclusively on morphological features, a task that requires a high level of expertise [5]–[7]. Later, a variety of genetic-based methods were used to complement and in some cases substitute for morphology-based approaches. These included the use of isoenzymes, randomly amplified polymorphic DNA (RAPD), restriction fragment length polymorphisms (RFLP), and the sequencing of nuclear genes [2]. More recently, mitochondrial protein-coding genes have been employed with increasing popularity due to their faster rate of evolution [8], [9].

The two most commonly used genes are apocytochrome *b* (*cob*) and cytochrome *c* oxidase subunit I (*COI*). The majority of the studies employing these markers focused on assessing the degree of molecular variation both at the inter- and intra-specific levels, identifying cryptic species and mapping genetic variability on sampling localities. A common finding of these analyses is a previously undetected high degree of genetic differentiation, leading to the speculation that ciliate diversity is much higher than morphology initially suggested [10]–[17].

However, in most of these studies, DNA was extracted from clonal cultures and sequences were obtained by sequencing PCR products directly. Thus, there was little insight into the degree of divergence of genes at the level of the individual cell, a process termed as heteroplasmy. Heteroplasmy has been documented in various metazoan groups [18]–[25] and the kinetoplastid protist *Trypanosoma cruzi* [26] but not in ciliated protists. Therefore, it is not possible to conclusively determine whether the observed intra-specific divergence of mitochondrial genes in ciliates is genuine or the result of intra- individual genetic variation. To that end, Barth *et al.* [13] reported a small amount of genetic divergence within single cells of *Paramecium caudatum*.

Here, we sought to characterize DNA polymorphisms at the *COI* locus at the individual, population and species levels in the genus *Paramecium*, a model organism that has lately been the focus of intense studies of genetic differentiation [13]–[17]. We examined a total of 147 clones from five species of *Paramecium*: *P. bursaria*, *P. duboscqui*, *P. neprhridiatum*, *P. caudatum* and *Paramecium* sp.. We found a small degree of intra-individual genetic divergence in all five species that could not be attributed to amplification, cloning and sequencing errors alone. Since presence of heteroplasmy may affect species delineation and result in skewed interpretations of phylogenetic and phylogeographic studies, we also investigated how the observed intra-individual divergence may affect the nature of such studies in *Paramecium*.

## Results

### Primary sequence analysis of the *COI* gene and genetic diversity

The amplified *COI* gene fragment of *Paramecium bursaria* is 812 bp, while that of *P. caudatum*, *P. duboscqui*, *P. nephridiatum*, and *Paramecium* sp. is 803 bp. The inter-specific genetic divergence ranges from 23.0% to 30.2% (Table S1–5 in file S1), while the intra-specific variation is 0.1%–10.9% (Table S1–5 in file S1). The pairwise sequence divergence for all five *Paramecium* species at the intra-individual level is less than 2%.

**Figure 1:**
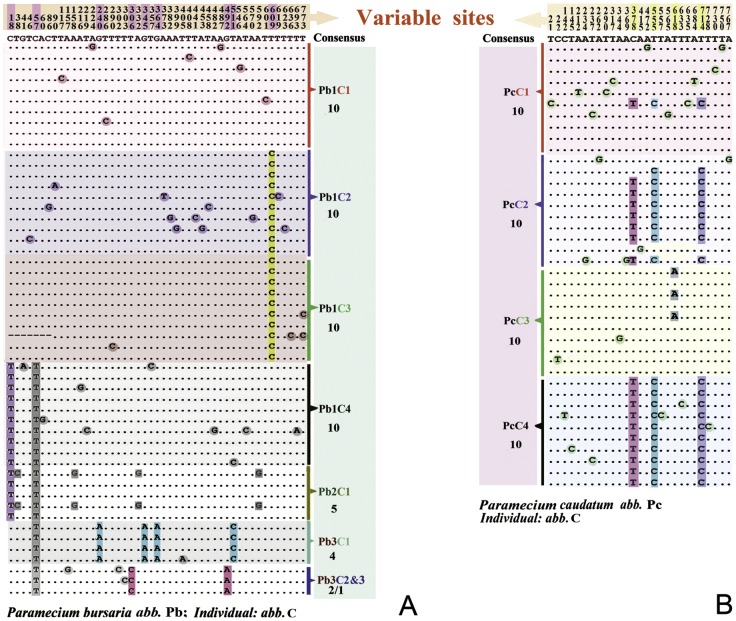
Variable site details of *Paramecium bursaria* and *Paramecium caudatum*. Alignment of amplified *COI* sequences (primer binding regions excluded) based on datasets *COI*_nb and *COI*_nc. The nucleotides shaded with rectangles and circles are used to illustrate the levels of diversity found among different clones.

All surveyed individuals bear more than one haplotype of the *COI* gene (Table S6 in file S2), with the exception of Pb3C3 for which only one clone is available (Table S7 in file S2, Fig. S1 in file S3). The number of variable sites is slightly higher than expected even after accounting for PCR, cloning and sequencing error rates (see also Experimental Procedures section) [27]. Synonymous substitutions are dominant especially in the cases of *P. bursaria* and *P. caudatum* (Table S7 in file S2). *P. bursaria* has 46 variable and 13 parsimony informative sites, nine of which (C/T_18_, C/T_57_, G/A_240_, T/C_306_, G/A_345_, G/A_364_, G/A_492_, T/C_511_ and T/C_609_, Fig. 1A) are identical at the individual level (Fig. S1A in file S3). There are 27, 9, 22 and 27 variable sites in *P. caudatum*, *Paramecium* sp., *P. nephridiatum* and *P. duboscqui*, respectively (Fig. 1B and Table S7 in file S2 and Figs. S1 B–E in file S3). Three parsimony informative sites are present in *P. caudatum* (C/T_378_, T/C_555_, T/A_618_, T/C_714_) and one in *Paramecium* sp. (G/A_195_), while none are found in *P. nephridiatum* and *P. duboscqui* (Fig. 1B and Figs. S1B–E in file S3). Interestingly, there is an in-frame deletion (sites 324, 325, 326) in *P. dubosqui* (PdC26, JX082135) (Fig. S4 in file S3).

### Haplotype network and geographic distribution

**Figure 2:**
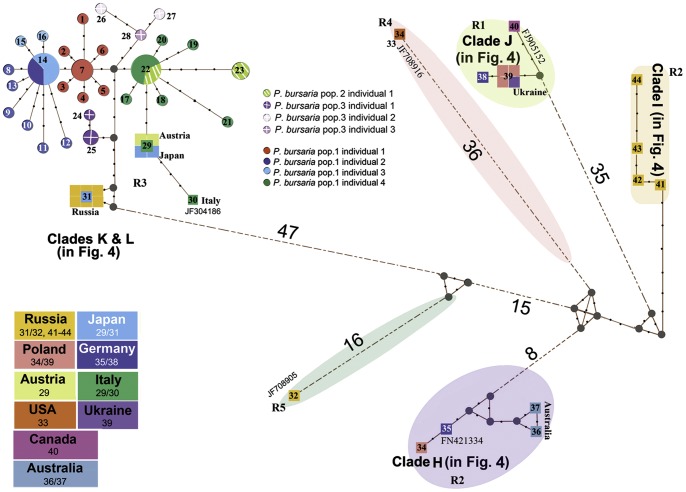
Haplotype network of *Paramecium bursaria* generated on the basis of the maximum-likelihood tree. Black circles indicate intermediate or unsampled haplotypes, while lines between points represent nucleotide substitutions. Wherever there are more than four substitutions, they are indicated by numbers. Clades K, L, H, I, J are marked to match the corresponding clades in Figure 4. Colored circles and squares indicate haplotypes whose size is proportional to the number of individuals showing that haplotype. Haplotype_7 is represented by 4 clones of Pb1C1; haplotype_14 is represented by 4 clones of Pb1C2 and 7 clones of Pb1C3; haplotype_22 is represented by 5 clones of Pb1C4 and 3 clones of Pb2C1; haplotype_25 is represented by 3 clones of Pb3C1.

A total of forty-four haplotypes were identified within *Paramecium bursaria*. Twenty eight of these (haplotypes_1-28) were generated in the present study, while 16 (haplotypes_29-44) are from previously sequenced individuals. Haplotypes PbCOI_14 and PbCOI_22 are dominant and shared by 11 clones of Pb1C2/3 and 8 clones of Pb1C4/Pb2C1 respectively (Fig. 2). In both network and tree analyses, the haplotypes are divided into five major clades which correspond very closely to syngen types of *P. bursaria* (R1–R5) as proposed by Greczek-Stachura *et al.* [16]. All *P. bursaria* haplotypes sequenced in this study belong to syngen R3 and group with isolates from Japan and Europe. For the most part there is no correlation between the clades and the geographic origins of the haplotypes in any of the syngens. The one exception is syngen R2 which shows a clear geographic separation between the isolates from Russia and those from the rest of Europe and Australia (Fig. 2 and Fig. S2 in file S3).

**Figure 3:**
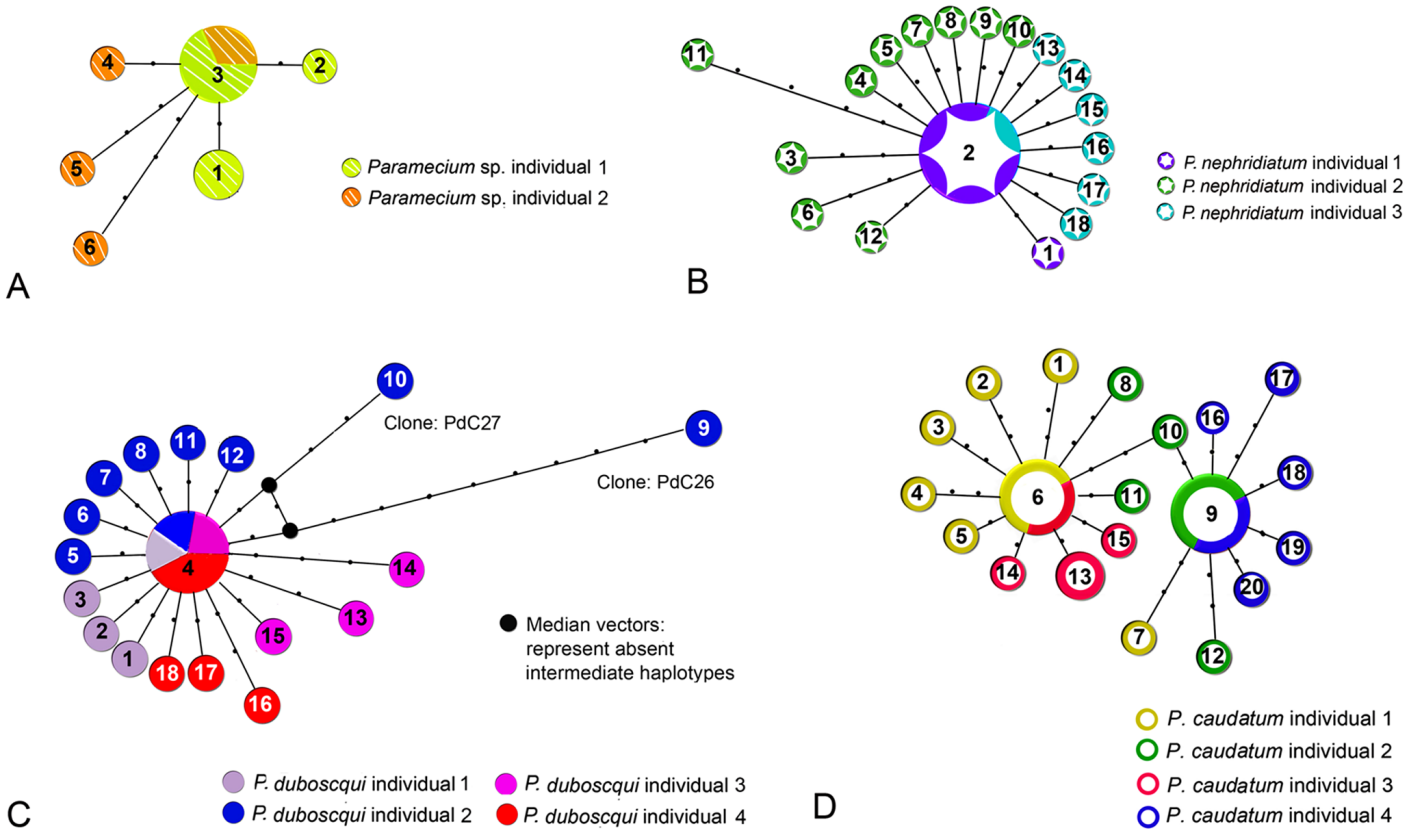
Haplotype network of *Paramecium* sp. (A) based on the dataset *COI*_nw, *P. nephridiatum* (B) based on dataset *COI*_nn, *P. duboscqui* (C), based on dataset *COI*_nd and *P. caudatum* (D) based on dataset *COI*_nc generated on the basis of the maximum-likelihood tree. Each line between points represents a single mutational step. A haplotype is represented by a circle whose size is proportional to the number of individuals showing that haplotype. Haplotypes are colored to match the respective population in the map. A) Haplotype_1 is represented by 2 clones of PwC1; Haplotype_3 is represented by 4 clones of PwC1 and 2 clones of PwC2; B) Haplotype_4 is represented by 1 clone of PdC1, 1 clone of PdC2, 1 clone of PdC3 and 2 clones of PdC4; C) Haplotype_2 is represented by 4 clones of PnC1 and 1 clone of PnC3; D) Haplotype_6 is represented by 4 clones of PcC1 and 5 clones of PcC3; Haplotype_9 is represented by 6 clones of PcC2 and 5 clones of PcC4; Haplotype_13 is represented by 3 clones of PcC3.

Six haplotypes are found within 12 clones of *Paramecium* sp. The dominant haplotype PwCOI_3 is shared by 6 clones of PwC1/C2 (Fig. 3A). Eighteen haplotypes are detected for 22 sequenced clones of *P. nephridiatum*. Haplotype PnCOI_2 is dominant and shared by 5 clones (Fig. 3B). The number of sequenced clones and haplotypes of *P. duboscqui* resemble those of *P. nephridiatum*. Haplotype PdCOI_4 is dominant and shared by 5 clones. Haplotypes PdCOI_9 and PdCOI_10 are the most divergent haplotypes of *P. duboscqui* (Fig. 3C). PcCOI_6 and PcCOI_9 are the dominant haplotypes of *P. caudatum* and shared by 9 (PcC1/3) and 11 clones (PcC2/4), respectively.

### Genetic structure analyses

AMOVA analyses indicate that genetic differentiation within individuals and populations is high (Φst values = 0.67, Φsc values = 0.56) and significant (*P*<0.00001). The analyses show that the majority of genetic variation is present among individuals within populations (sum of squares = 38.414, Table S8 in file S2).

### Phylogenetic analyses based on *COI* gene sequences

**Figure 4:**
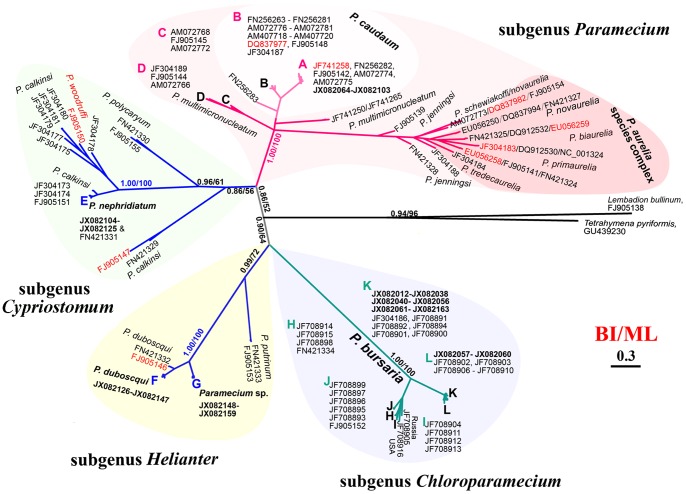
Phylogenetic tree of the barcoding region of 263 cytochrome *c* oxidase subunit I (*COI*) gene sequences of the genus *Paramecium* and genera *Lembadion* and, *Tetrahymena* inferred by Bayesian Inference (BI) analysis based on dataset *COI*-f. The branches are shaded according to subgenera *Chloroparamecium*, *Helianter*, *Cypriostomum*, *Paramecium*, proposed by Fokin *et al.* [28]. The scale bar corresponds to 30 substitutions per 100 nucleotide positions. For *P. bursaria*, Clade H includes populations sampled from Australia, Germany, and Poland; Clade I and J include populations sampled from Russia and Poland, Germany, Ukraine, and Canada; Clade K includes populations sampled from China (Pb1C1-4 & Pb2C &Pb3C2-3), Austria, Japan, and Italy; Clade L includes populations sampled from China (Pb3C1), Russia, and Japan (see details in Fig. S2 in file S3). For *P.caudatum*, Clade A includes populations sampled from China (PcC1-4 and AM072774), Australia, USA, and Brazil while members of Clade B were sampled from Germany, Italy, Russia, UK, Norway, Hungary, Slovenia, and Austria (see details in Fig. S3 in file S3). Inconsistent sequences (FJ905146, FJ905147, EU056259, EU056258, DQ837977, DQ837982, JF741258, JF304183) are marked in red [14], [42], [47].

All *Paramecium COI* sequences cluster in four distinct clades corresponding to four subgenera (*Paramecium*, *Cypriostomum*, *Helianter* and *Chloroparamecium*) as those are described by Fokin *et al.* [28] (Fig. 4). In the clade of subgenus *Paramecium*, isolates of *P. caudatum* group into three sister clades (A, B and FN256283). Sequences of *P. multimicronucleatum* form three clades (C, D and JF741250/JF741265) and are not monophyletic. *Paramecium jenningsi* and *P. schewiakoffi* fall into the *P. aurelia* complex clade. Among the four species of the subgenus *Cypriostomum*, *P. nephridiatum* and *P. polycaryum* are recovered as monophyletic. However, sequence FJ905150 designated as *P. woodruffi* falls into the *P. calkinsi* clade. Species of the subgenus *Helianter* are monophyletic. Our phylogenetic analyses suggest that *Paramecium* sp. is a previously unidentified member of the *Helianter* clade, which is more closely related to *P. duboscqui* than to *P. putrinum*. The *Chloroparamecium* clade contains only *P. bursaria* sequences that are separated into two branches (K-L and I-H-J) (Fig. 4). In all cases, the cloned sequences from all individuals of the same species cluster together, thus no aberrant sequences were seen on the tree.

## Discussion

### Heteroplasmy in *Paramecium* spp

While mtDNA heteroplasmy is well documented in metazoan species [18]–[25], such is not the case in protists. Recently, heteroplasmy was reported for the first time in the mitochondrial genome of the kinetoplastid protist *Trypanosoma cruzi* [26]. In the present study we assessed diversity at the *COI* locus in five species of *Paramecium*; *P. bursaria*, *P. duboscqui*, *P. nephridiatum*, *P. caudatum* and *Paramecium* sp. We found multiple *COI* haplotypes in all five *Paramecium* spp. The level of intra-species haplotype divergence ranged between 0.1–1.3%, lower than typical metazoan heteroplasmy which ranges between 2–6% [29]. Though we tried to account for PCR and cloning errors in our estimations, it is still possible that some of the detected haplotypes are indeed slippage errors, especially those that have only a few polymorphisms. However, in several cases a slight divergence was noted even after such errors had been accounted for. Since there are thousands of mitochondria within single ciliated cells, it is very likely that the observed divergence is derived from the presence of diverse copies of the mitochondria within a single cell. Our results resemble those of Barth *et al.* who detected a few polymorphic sites in single cells of *P. caudatum* [13]. Regrettably, the exact number of these sites was not reported in their study.

Another plausible explanation is that these are cases of micro-heteroplasmy, which corresponds to the presence of multiple haplotypes in an individual due to independent mutations [29], [30]. These haplotypes occur at a low frequency and are polymorphic in the protein-coding region. Evidence for micro-heteroplasmy comes from humans and grasshoppers but has not been studied as much in other groups. This is mainly because in studies of heteroplasmy, haplotypes with less than 2% divergence are usually dismissed.

An alternate explanation for the presence of such an abundance of haplotypes is that they represent nuclear encoded mitochondrial pseudogenes (NUMTs) [31]–[34]. These are non-functional copies of mitochondrial genes that have been incorporated into the nuclear genome and bear varying degrees of similarity to the “true” mitochondrial genes. NUMTs are found in the genomes of metazoans and plants but less so in protists [11], [29], [35]–[39]. NUMTs are easily distinguished when they contain stop codons, indels and a high number of non-synonymous substitutions in the coding sequence. In this study we identified one sequence that contained an in-frame deletion and a few sequences with a high number of non-synonymous substitutions, features indicative of NUMTS.

A less common source for the observed level of intra-individual diversity is the presence of gene duplications [40], [41]. A number of ciliate mitochondrial genomes have now been sequenced and gene duplications are not that frequent [32], [42]–[44]. The *rnl* gene is duplicated in *Tetrahymena* spp. and *nad9* in *Tetrahymena thermophila* and *T. malaccensis* [32]. The mitochondrial genomes of *Paramecium caudatum* and *P. tetraurelia* have also been sequenced and there is no evidence of gene duplications [32], [42]. While it is not known if such duplications occur in other *Paramecium* species, it seems that this option though theoretically possible is in fact highly unlikely.

### Is the *COI* gene a suitable marker for discriminating between *Paramecium* spp.?

*Paramecium* is a species-rich genus comprising many closely related species that can be mostly discriminated with mating tests and SSU gene sequences [28], [45], [46]. Some studies have employed the *COI* gene to assess genetic diversity and delineate the various *Paramecium* species [47], [48]. In this study, the genus *Paramecium* is monophyletic, as are the four subgenera described by Fokin *et al.* [28]. The general topology of the *COI* tree resembles that of the SSU tree [28], [46]. All the species-specific sequences cluster together and there is no haplotype sharing among the examined species (Fig. 4 and Figs. S2–3 in file S3). Thus, we found no evidence that the slight intra-individual variation influenced phylogenetic relationships in any way. Furthermore, the boundary between the intra- and inter-specific genetic distances among all surveyed *Paramecium* taxa is maintained. The presence of such a gap has been termed the “barcoding gap” and it is the key discriminator in the *COI*-barcode approach [8], [9], [47]. In the present study, the intra-specific genetic divergence of *COI* ranged from 0.1% to 10.9% and the inter-specific was >23% (Tables S1–2 in file S1), similar to previous estimates in other *Paramecium* species [13]–[16], [47], [48].

The degree of genetic divergence within *Paramecium* is higher than that observed in other ciliates. Consequently, thresholds for species delimitation differ among ciliate taxa; ∼1% for *Tetrahymena* and <18% for *Carchesium* [10], [49]–[51]. Hence, levels of intra-specific variability in ciliates are genus specific and should be determined empirically [47]. The database for *Paramecium COI* is becoming increasingly more comprehensive and is second only to *Tetrahymena*. Thus, assignment of new, unknown species to at least the subgenus level is now possible. Furthermore, spotting incongruities in the *Paramecium* tree is becoming gradually more feasible. For instance, sequences that were previously identified as *P. novaurelia* and *P. multimicronucleatum* seem to be *P. caudatum* instead [14], [52]. Therefore, we recommend that in order to avoid such drawbacks, researchers should include more than just isolates of the group of interest in phylogenetic analyses so that discordant sequences become obvious.

### Genetic diversity and geographic distribution

Our population-level analyses in *Paramecium bursaria* and *P. caudatum* indicate that intra-individual variation has only a minor effect. In most cases, the parsimony networks exhibit star-like topologies, suggesting that the detected polymorphisms are not shared among haplotypes (Figs. 2, 4, Figs. S2, S3 in file S3). Haplotype sharing between two temporal isolates of *P. bursaria* (i.e. sampled at different time points in the same location) was limted (Fig. 2, Table S6 in file S2, Fig. S2 in file S3), warranting further investigation for spatio-temporal haplotype shifts. All *P. bursaria* haplotypes sequenced in this work belong to syngen R3 (Figs. 2, 4, Fig. S2 in file S3). Haplotypes from the various syngens show no geographic partitioning. Syngen R2 is the exception and divided into two subclades: one containing samples from Russia and the other containing an assortment of samples from Europe and Australia. Thus far, syngen R3 is the most densely sampled and it is also the most widely distributed. We cannot speculate about the distribution of R4 and R5 since they consist of only a handful of samples.

In *P. caudatum* all new haplotypes form a distinct clade with isolates from the American continent and Australia (Fig. S3 in file S3). Thus, this clade is more widespread than the second *P. caudatum* clade that so far consists of isolates exclusively from Europe.

Taken together, these results suggest that gene flow has been maintained in the widely distributed clades of both *P. bursaria* and *P. caudatum*. In ciliate taxa, both cosmopolitan and endemic distributions have been shown [13], [16], [17], [53]–[55]. However, most studies focus on relatively well-studied taxa mainly those of the genera *Paramecium* and *Tetrahymena*. Thus, it would be informative to examine genetic diversity and its geographic distribution in other, less studied taxa.

## Materials and Methods

### Ethics Statement

We confirm that no specific permits were required for the described field studies. None of the sampling localities are privately-owned or protected in any way. Endangered or protected species were not involved.

### Sample collection and culturing

For the purpose of this study, we use the term population to define individuals of the same species that were collected from the same locality and at the same time. Cells of *Paramecium caudatum* were picked from an established culture that has been maintained since 2009. Individuals of *P. bursaria*, *P. nephridiatum*, *P. duboscqui* and *Paramecium* sp. were sampled from different biotopes in Qingdao, China (Table S6 in file S2 and Fig. S5 in file S3). *P. bursaria* was collected at two separate time points and localities. Plastic traps were immersed in water and then transferred to the laboratory where cell picking occurred within 3–5 days.

Identification of all species was based on morphological features observed with live microscopy, staining and SSU sequences (data available upon request) [56], [57].

### DNA extraction, PCR amplification and sequencing

Single cells were isolated and washed several times in sterile water (fresh water was used for fresh water isolates and marine water for brackish isolates) to remove potential minute sized protists, and subsequently transferred to 1.5 ml microtubes with a minimum volume of water. Total genomic DNA was extracted from single cells with the DNeasy & Tissue Kit (Shanghai, QIAGEN China, China) according to manufacturer's specifications with the following modification: only 1/16 of the suggested volume was used for each step. The concentration and quality of the extracted genomic DNA was verified using the NanoDrop-ND1000 spectrophotometer.

A fragment of about 800 bp of mitochondrial *COI* gene was amplified using primers F388dT and R1184dT [47]. Amplification cycle conditions were as follows: 4 min at 94°C followed by 35 cycles at 94°C for 45 s, 60°C for 75 s, 72°C for 90 s, and a final extension at 72°C for 10 min. The polymerase reaction was performed with TAKARA high fidelity *Ex Taq™* polymerase, with an error rate of 2×10^−6^ per nucleotide per cycle, (http://www.clontech.com/takara). Thus, the error for each of our fragments is estimated to about 0.06 nucleotides. Following PCR amplification, the resulting amplicons were purified using the Spin Column PCR Product Purification Kit (Sangon Code: SK1132, Sangon Bio. Co., China) according to manufacturer's specifications. The purified PCR products were then inserted into the pMD®19-T simple vector (TaKaRa Code: D104A, Takara Bio Technology Co., Ltd., Dalian, China) and bi-directionally sequenced with the amplification primers in Beijing Genomics Institute (BGI) division in Qingdao [58]–[60].

### Sequence analyses

For this study, a total of 147 new *COI* sequences were generated. The sequence fragments were imported into the Geneious software program, version 5.1 [61] and assembled into contigs. The sequences were then checked by eye to remove ambiguous base calls and rule out sequencing errors in AT rich regions. Ambiguous, low quality bases were trimmed from both the 5′ and 3′ regions. Subsequently, all of the newly generated sequences were aligned with ClustalW as implemented in BioEdit v7.1.3 [62], and then manually refined in MEGA v5.0 based on the predicted amino acid sequences [32], [42], [63]. In addition, we included all *Paramecium* species *COI* sequences available in GenBank (up until March, 2012). Eight alignment datasets were created for the subsequent analyses (Table S1 in file S1).

The variable sites from the datasets *COI*_nb, *COI*_nc, *COI*_nn, *COI*_nd and *COI*_nw were imported into Geneious v5.1 [61] and the genetic similarity and coverage information were estimated (Fig. S1 in file S3). Percent identity of the various sequences was calculated with the Geneious v5.1 software [61].

### Haplotype searching and genetic structure analyses

Networks depicting the distribution and relationships among haplotypes of *P. bursaria*, *P. caudatum*, *P. nephridiatum*, *P. duboscqui* and *Paramecium* sp. were derived by using the median-joining methods as implemented in the program Network 4.6.1.0 (http://www.fluxus-engineering.com/) [64].

The software program ARLEQUIN 3.11 was used to perform a hierarchical analysis of molecular variance (AMOVA) in order to quantify genetic structure of *Paramecium bursaria* within and among individuals and populations [65]. Sequences were grouped first by individual and then by population.

### Phylogenetic analyses

The final alignment of dataset *COI*_f contained 262 taxa and 813 positions representing the entire ciliate barcoding region as defined by Strüder-Kypke and Lynn [47]. Sequences shorter than 400 bp were not considered for further analyses. Protein domain analysis revealed that these sequences only included the ciliate intron region of *COI*.

The GTR+Γ+I model was selected by Modeltest v.3.7 [66], and the reliability of internal branches was assessed using a non-parametric bootstrap method with 1000 replicates. Bayesian inference (BI) analysis was performed with MrBayes v3.1.2 [67] using the GTR+Γ+I model selected by MrModeltest 2 [68] under the AIC criterion. Two parallel runs were performed over 8,000,000 generations with every 100^th^ tree sampled. The first 20,000 trees were discarded as burn-in. The maximum posterior probabilty was determined out of the sampled trees, approximating it with the Markov Chain Monte Carlo (MCMC). Posterior probabilities (PP) of all branches were calculated using a majority-rule consensus approach. Phylogenetic trees were viewed and edited with MEGA 5 [63].

### Nucleotide sequences accession numbers

The new nucleotide sequences have been submitted to GenBank. The corresponding accession numbers are JX082012–JX082038, JX082040–JX082159 (Table S6 in file S2).

## Supporting Information

File S1 **Table S1, The datasets analyzed in the present investigation. Table S2, The sequence similarity of Parmecium bursaria (including 29 populations from 11 countries). Table S3, The sequence similarity of Parmecium caudatum (including 38 populations from 15 countries). Table S4, The sequence similarity of Parmecium sp., P. duboscqui and P.nephridiatum (including newly sequenced sequences). Table S5, The sequence similarity of the newly sequenced species.**
(XLS)

File S2 **Table S6, Specifications of** ***Paramecium*** **species clone generated in the present work. Table S7, Details of the synonymous and non- synonymous mutation information of each individual infered from amino acid analysis. Table S8, Hierarchical AMOVA analyses of the** ***COI*** **gene of** ***Paramecium bursaria*** **, P-values with the star marks show the significance.**
(DOC)

File S3 **Figure S1, Sequence alignments of variation in different clones of** ***Paramecium bursaria*** **(A,** ***COI*** **_nb),** ***P. caudatum*** **(B,** ***COI*** **_nc),** ***Paramecium*** **sp. (C,** ***COI*** **_nw),** ***P. duboscqui*** **(D,** ***COI*** **_nd) and** ***P. nephridiatum*** **(E,** ***COI*** **_nn). Figure S2, The blowup of details of the tree showing** ***Paramecium bursaria*** **relationships in** **figure 4** **.Figure S3, The blowup of details of the tree showing** ***Paramecium caudatum*** **relationships in** **figure 4** **. Figure S4, Details of sequence alignments of variation in different clones of** ***P. duboscqui*** **, and the novel three deletion positions were marked in red. Figure S5, The sample localities in the present work.**
(DOC)
