# Supplementary material for: Genetic Differentiation of the Mitochondrial Cytochrome Oxidase c Subunit I Gene in Genus Paramecium (Protista, Ciliophora)
Source: PLoS One. 2013 Oct 29;8(10):e77044. doi: 10.1371/journal.pone.0077044 (PMC3812207; doi:10.1371/journal.pone.0077044)
Supplement: File S2 — Table S6, Specifications of Paramecium species clone generated in the present work. Table S7, Details of the synonymous and non- synonymous mutation information of each individual infered from amino acid analysis. Table S8, Hierarchical AMOVA analyses of the COI gene of Paramecium bursaria , P-values with the star marks show the significance. (DOC) [file pone.0077044.s002.doc]

**Table S6.** Specifications of *Paramecium* species clone generated in the present work. *Paramecium bursaria* abb. Pb*; P. caudatum* abb. Pc; *P. nephridiatum* abb. Pn; *P. duboscqui* abb. Pd; *Paramecium* sp. abb. Pw.

| **Species** | **Cells**  **abbreviation** | **Sample locality** | **Sample date** | **No. of**  **clones** | **No. of**  **haplotypes** | **Overall** | **GenBank**  **Accession Numbers** |  |
| --- | --- | --- | --- | --- | --- | --- | --- | --- |
| ***P. bursaria*** | Pb1C1 | China, Qingdao  Zhongshan park;  Fresh water;  36°06´N, 120°35´E | March 15^th^, 2011 | 10 | 7 | 28 | JX082012-JX082021 |  |
|  | Pb1C2 |  |  | 10 | 7 |  | JX082022- JX082031 |  |
|  | Pb1C3 |  |  | 9 | 4 |  | JX082032- JX082038  JX082040- JX082041 |  |
|  | Pb1C4 |  |  | 10 | 6 |  | JX082042- JX082051 |  |
| ***P. bursaria*** | Pb2C1 | China, Qingdao  Zhongshan park;  Fresh water;  36°06´N, 120°35´E | April 26^th^, 2011 | 5 | 2 |  | JX082052- JX082056 |  |
| ***P. bursaria*** | Pb3C1 | China, Qingdao;  Laoshan mountain; Fresh water; 36°21´N, 120°60´E | May 28^th^, 2011 | 4 | 2 |  | JX082057- JX082060 |  |
|  | Pb3C2 |  |  | 2 | 2 |  | JX082061- JX082062 |  |
|  | Pb3C3 |  |  | 1 | 1 |  | JX082063 |  |
| ***P. caudatum*** | PcC1 | China, Qingdao;  Zhongshan park  Fresh water;  36°08´N, 120°43´E | cultured since 2009 | 10 | 7 | 20 | JX082064- JX082073 |  |
|  | PcC2 |  |  | 10 | 5 |  | JX082074- JX082083 |  |
|  | PcC3 |  |  | 10 | 4 |  | JX082084- JX082093 |  |
|  | PcC4 |  |  | 10 | 6 |  | JX082094- JX082103 |  |
| ***P. nephridiatum*** | PnC1 | China, Qingdao coast  brackish water,  36°05´N, 120°30´E | March 6^th^, 2011 | 5 | 2 | 18 | JX082104-JX082109 |  |
|  | PnC2 |  |  | 10 | 10 |  | JX082110-JX082119 |  |
|  | PnC3 |  |  | 7 | 7 |  | JX082119-JX082125 |  |
| ***P. duboscqui*** | PdC1 |  |  | 4 | 4 | 18 | JX082126- JX082129 |  |
|  | PdC2 |  |  | 9 | 9 |  | JX082130-JX082138 |  |
|  | PdC3 |  |  | 4 | 4 |  | JX082139-JX082142 |  |
|  | PdC4 |  |  | 5 | 4 (PdCOI_4, 16-18) |  | JX082143-JX082147 |  |
| ***Paramecium* sp.** | PwC1 |  |  | 7 | 3 (PwCOI_1-3) | 6 | JX082148- JX082154 |  |
|  | PwC2 |  |  | 5 | 4 (PwCOI_3-6) |  | JX082155-JX082159 |  |

**Table S7. Details of the synonymous and non- synonymous mutation information of each individual infered from amino acid analysis.**

| **Species** | **Cells**  **abbreviation** | **No. of parsimony**  **informative sites** | **No. of**  **synonymous** | **No. of**  **non-synonymous** | **No. of**  **variable sites** |
| --- | --- | --- | --- | --- | --- |
| *P. bursaria* | Pb1C1 | 13 | 12 | 4 | 46 |
|  | Pb1C2 |  | 15 | 6 |  |
|  | Pb1C3 |  | 9 | 2 |  |
|  | Pb1C4 |  | 14 | 2 |  |
|  | Pb2C1 |  | 9 | 4 |  |
|  | Pb3C1 |  | 10 | 0 |  |
|  | Pb3C2 |  | 10 | 2 |  |
|  | Pb3C3 |  | 9 | 0 |  |
| *P. caudatum* | PcC1 | 4 | 7 | 7 | 27 |
|  | PcC2 |  | 4 | 2 |  |
|  | PcC3 |  | 4 | 2 |  |
|  | PcC4 |  | 6 | 3 |  |
| *P. nephridiatum* | PnC1 | 0 | 0 | 1 | 22 |
|  | PnC2 |  | 4 | 11 |  |
|  | PnC3 |  | 3 | 3 |  |
| *P. duboscqui* | PdC1 | 0 | 1 | 2 | 27 |
|  | PdC2 |  | 5 | 10 |  |
|  | PdC3 |  | 3 | 3 |  |
|  | PdC4 |  | 1 | 2 |  |
| *Paramecium* sp. | PwC1 | 1 | 3 | 0 | 9 |
|  | PwC2 |  | 3 | 3 |  |

**Table S8.** Hierarchical AMOVA analyses of the *COI* gene of *Paramecium bursaria*, P-values with the star marks show the significance.

| Hierarchical  structure | Source  of variation | Sum of  Squares (df) | Variance  components | P | Fixation  index |
| --- | --- | --- | --- | --- | --- |
| 1 | Among Populations | 24.013 (2) | 0.64967 | >0.05 | Φ_CT_ = 0.26 |
| 2 | Among Individuals  Within Populations | 38.414 (5) | 1.01068 | <0.00001* | Φ_SC_ = 0.56 |
| 3 | Within Individuals | 35.650 (44) | 0.81023 | <0.00001* | Φ_ST_ = 0.67 |
|  | Total | 98.077 (51) | 2.47058 |  |  |
