# Supplementary material for: Genetic Differentiation of the Mitochondrial Cytochrome Oxidase c Subunit I Gene in Genus Paramecium (Protista, Ciliophora)
Source: PLoS One. 2013 Oct 29;8(10):e77044. doi: 10.1371/journal.pone.0077044 (PMC3812207; doi:10.1371/journal.pone.0077044)
Supplement: File S3 — Figure S1, Sequence alignments of variation in different clones of Paramecium bursaria (A, COI _nb), P. caudatum (B, COI _nc), Paramecium sp. (C, COI _nw), P. duboscqui (D, COI _nd) and P. nephridiatum (E, COI _nn). Figure S2, The blowup of details of the tree showing Paramecium bursaria relationships in figure 4 .Figure S3, The blowup of details of the tree showing Paramecium caudatum relationships in figure 4 . Figure S4, Details of sequence alignments of variation in different clones of P. duboscqui , and the novel three deletion positions were marked in red. Figure S5, The sample localities in the present work. (DOC) [file pone.0077044.s003.doc]

**Supplementary 9: Figure S1 - Sequence alignments of variation in different clones of *Paramecium bursaria* (A, *COI_*nb), *P. caudatum* (B, *COI*_nc), *Paramecium* sp. (C, *COI*_nw), *P. duboscqui* (D, *COI*_nd) and *P. nephridiatum* (E, *COI*_nn).** The graphs were constructed with the alignment viewer program Geneious v5.1 (Drummond, et al. 2010). The identities of each residue are marked as different height bars at the bottom of each graph – the higher the bar, the higher the identity. There are 46 variable sites within 51 clones of *P. bursaria*；27 variable sites of 40 clones of *P. caudatum*；9 variable sites within 12 clones of *Paramecium* sp.; 27 variable sites of 22 clones of *P. duboscqui* and 22 variables sites of 22 clones of *P. nephridiatum*.

**Supplementary 10: Figure S2 - The blowup of details of the tree showing *Paramecium bursaria* relationships in figure 4.**

**Supplementary 11: Figure S3 - The blowup of details of the tree showing *Paramecium caudatum* relationships in figure 4.**

**Supplementary 12: Figure S4 - Details of sequence alignments of variation in different clones of *P. duboscqui*,** and the novel three deletion positions were marked in red.

**Supplementary 13: Figure S5 - The sample localities in the present work.** A represents Qingdao Coast, brackish water; B represents Zhongshan park, fresh water; C represents Laoshan Mountain, freshwater.


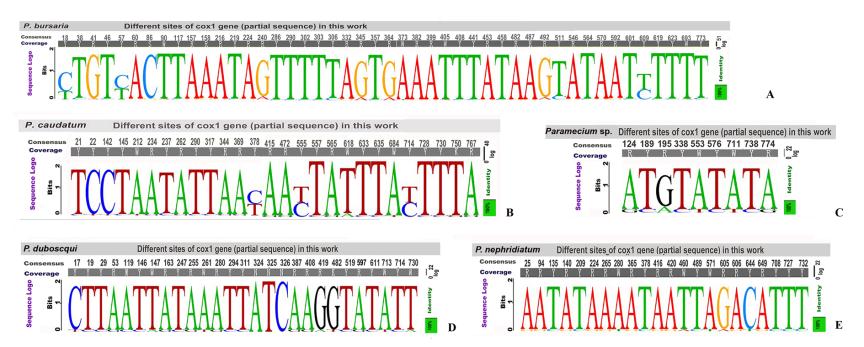


Fig. S1


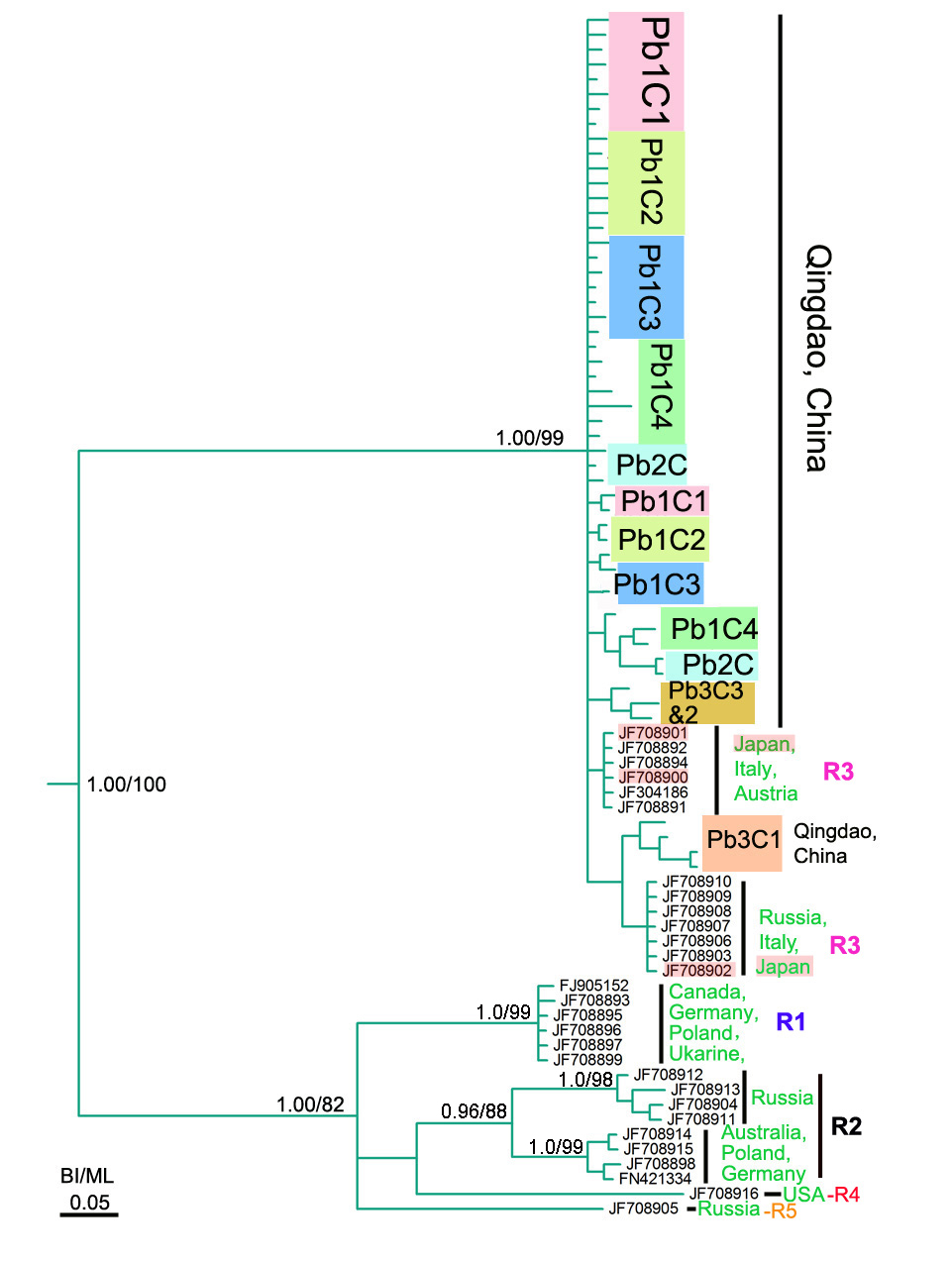


Fig. S2


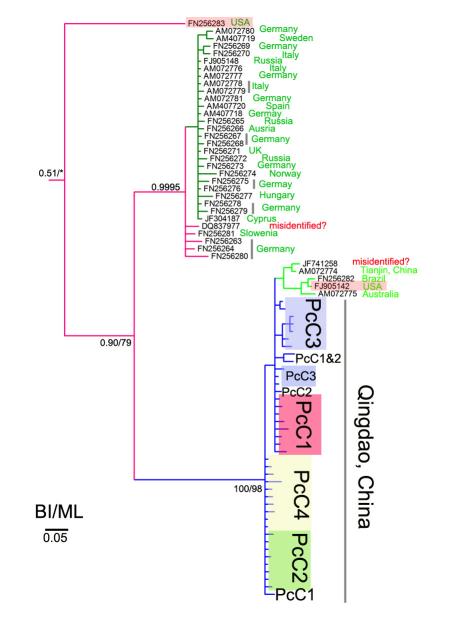


Fig. S3


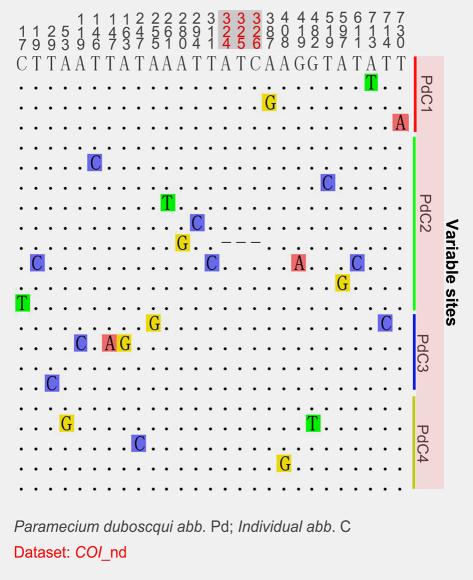


Fig. S4


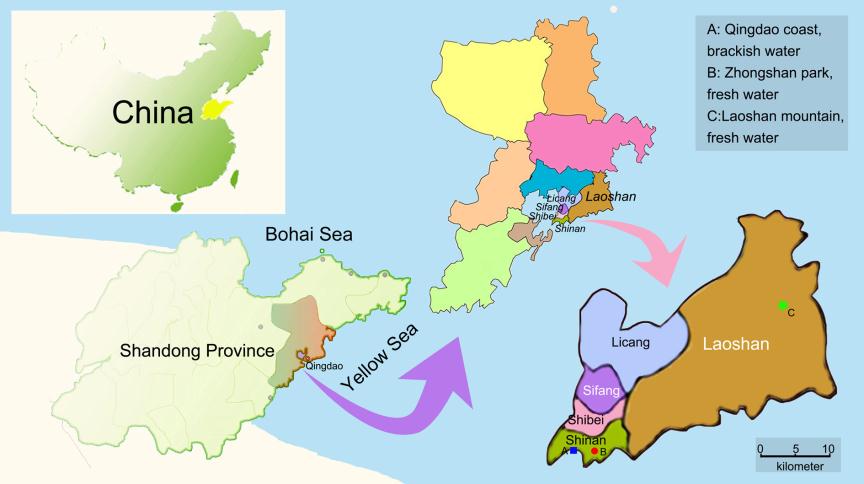


Fig. S5
